# Examining the Effectiveness of Telephone Clinics: A Review of a Telephone Foot and Ankle Orthopaedic Clinic in a District General Hospital in the United Kingdom

**DOI:** 10.7759/cureus.49196

**Published:** 2023-11-21

**Authors:** Surya Prasad, Eleni Cullen, Charlie Jowett

**Affiliations:** 1 Orthopaedics, The York Hospital, York and Scarborough Teaching Hospitals NHS Foundation Trust, York, GBR

**Keywords:** covid-19, follow-up appointment, video telemedicine, reduce cost, hospital footfall, infectious spread, outpatients clinic, orthopaedic clinic, patients satisfaction, telephone consultation

## Abstract

Introduction: Since the outbreak of COVID-19, significant value has been placed on preventative methods for pathogen spread. One such method is the use of telemedicine via telephone clinics (TC). This article is designed to study TC in a District General Hospital in the United Kingdom.

Aims: This clinical audit aims to evaluate the use of a TC in the United Kingdom and assess its effectiveness. It also aims to assess the use of a clinician-led triaging system to select patients who would receive subsequent telephone follow-ups (TFU).

Methodology: Two cycles were conducted. The first cycle was conducted in 2020 and the second cycle in 2022. In between the two cycles, a clinician-led triage system was implemented to reduce the number of patients being called back for a face-to-face (FTF) appointment. Data was collected regarding the outcomes of the appointments and compared between the two cycles. Chi-squared test statistical test was employed with a p-value < 0.05 deeming significance.

Results: Overall, more discharges were made for 2022 outcomes compared to 2020 outcomes (30% vs 19%; p = 0.03) after employing the clinician-led triage. The number of patients listed for a TFU increased when comparing the 2020 versus 2022 datasets (21% vs 12%; p = 0.026), and the overall number of patients not attending appointments decreased when comparing the 2020 versus 2022 datasets (9% vs 17%; p = 0.033).

Conclusion: This article shows that for this particular clinic, an orthopaedic clinician-led triaging system allows for a greater number of patients to be discharged, lessened need for FTF consultations, and increased adherence to appointments by patients. However, much work is yet to be done concerning the long-term consequences and issues of rolling out nationwide telemedicine.

## Introduction

COVID-19 has shaped how we view healthcare and its delivery to the general public [1]. Preventative methods for pathogen spread are crucial, and reducing footfall through hospitals during COVID-19 has been an invaluable aim through the use of telephone clinics (TC). In the hospital setting, there has been a move toward outpatient clinic appointments being conducted over the telephone [2]. This article aims to assess the use of TC through the use of a Foot and Ankle Orthopaedic Clinic run in the York District General Hospital in reducing patient footfall. This article studies how to streamline patient selection for telephone follow-up to reduce patients returning for face-to-face (FTF) appointments with a clinician-led triaging system.

## Materials and methods

The first cycle of research was conducted between 1st August and 1st December 2020. The inclusion criteria pulled all patients who attended the Foot and Ankle Clinic in The York General Hospital who received a telephone appointment in this timeframe. The total number of patients included in the first cycle was 103. These were consultant-led clinics flanked with specialty registrar support. The local information technology system was utilised to extract the outcome of the consultation, and the data was analysed using Microsoft Excel. The use of an orthopaedic clinician-led triage was then implemented to choose only particular patients for TC to reduce the number of patients having to return for FTF appointments.

The second cycle was conducted between 1st August and 1st December 2022 with a similar methodology. Patients having a simple preoperative assessment over the telephone were excluded. The letters that were uploaded to the internal hospital computer system and sent to each patient were analysed for any discrepancies in coding the outcome of the appointment. The total number of patients included in this cycle was 147. The data was split into trauma and elective patients and further divided into initial versus follow-up consults for valid comparisons to be drawn.

The outcomes of the consultations were split into those who could be discharged, those who needed further follow-up over the telephone, and those who needed further FTF follow-up. Data regarding scans were collected to assess the value of TC in making decisions for further radiological imaging. The data was compared across the same months of the calendar year to minimise the seasonal variation of footfall through the hospital. The same Wednesday/Thursday clinic was utilised to mitigate timing bias. Further data was collected about patient contact details to allow future studies on patient satisfaction and the rationale of non-attenders to further improve the delivery of care. The data points are patient demographics, diagnoses, clinic and appointment details, outcome of appointment, further referral needed, investigations required, and additional notes documented. The data was analysed within Microsoft Excel (Microsoft Corp., Redmond, Washington). For statistical analysis, the Chi-square test was employed with a p-value < 0.05 dictating a significant difference in the outcome.

## Results

The results are split into trauma versus elective consults. Table 1 presents a comparison between the datasets of initial trauma patients under the 2022 clinician-led triage system and the 2020 pre-triage system. The data is presented as N (%) in each column, with subsequent p-values calculated using the Chi-square test, and the p-values < 0.05 were considered significant. The percentages are shown to the nearest whole percentage point. There was some overlap in the patient categories, for example, some patients who did not attend (DNA) were subsequently discharged.

**Table 1:** Trauma patients' outcomes 2020 versus 2022 DNA: Did not attend.

Category	2020 outcomes, N (%)	2022 outcomes, N (%)	p-value
Total number of patients	48 (100%)	80 (100%)	-
DNA	8 (17%)	7 (9%)	0.089
Scans requested following the initial consult	3 (6%)	12 (15%)	0.068
Further face-to-face follow-up	17 (35%)	30 (38%)	0.406
Discharged	16 (33%)	33 (41%)	0.186
Further phone consult	7 (15%)	16 (20%)	0.220

Follow-up elective outcomes comparing 2020 versus 2022 are highlighted in Table 2. There was a reduction in DNA (7% vs 8%) category, fewer patients needed further FTF (49% vs 64%) follow-ups, and more patients were discharged (18% vs 10%). The data is presented as N (%) in each column, with subsequent p-values calculated using the Chi-square test, and the p-values < 0.05 were considered significant. The percentages are shown to the nearest whole percentage point.

**Table 2:** Follow-up elective outcomes 2020 versus 2022 DNA: Did not attend.

Categories	2020 outcomes, N (%)	2022 outcomes, N (%)	p-value
Total number of patients	39	61	-
DNA	3 (8%)	4 (7%)	0.414
Further face-to-face follow-up	25 (64%)	30 (49%)	0.072
Discharged	4 (10%)	11 (18%)	0.144
Listed for an operation	2 (5%)	4 (7%)	0.385
Further phone consult	5 (13%)	20 (33%)	0.012

The overall outcomes for each period are shown in Table 3. This table shows that only fewer FTF follow-ups are needed for 2022 outcomes (40% vs 50%), there is an increase in the number of patients listed for an operation (4% vs 2%), and there is an increase in the overall discharge rate (30% vs 19%). The data is presented as N (%) in each column, with subsequent p-values calculated using the Chi-square test, and the p-values < 0.05 were considered significant. The percentages are shown to the nearest whole percentage point.

**Table 3:** Comparing overall outcomes 2020 versus 2022

Categories	2020 outcomes, N (%)	2022 outcomes, N (%)	p-value
Total number of patients	103	147	-
Did not attend	17 (17%)	13 (9%)	0.033
Scans requested following the initial consult	5 (5%)	18 (12%)	0.023
Further face-to-face follow-up	52 (50%)	59 (40%)	0.053
Discharged	20 (19%)	44 (30%)	0.030
Listed for an operation	2 (2%)	6 (4%)	0.172
Further phone consult	12 (12%)	31 (21%)	0.026

## Discussion

The results of this study show that, for this particular clinic and this particular period, an orthopaedic clinician-led triaging system allows for a greater number of patients to be discharged (30% vs 19%; p = 0.030), lessened need for FTF consults, although not statistically significant (40% vs 50%; p = 0.053), and increased adherence to appointments by patients (DNA decreased from 17% to 9%; p = 0.033). One notable outcome is that the number of scans requested increased with a clinician-led triage system in place (12% vs 5%; p = 0.023). Clinical experience, judgement, and past exposures to repeat pathologies may explain why clinicians are likely to know which group of patients need FTF consultations. Criteria used to assess whether a patient needs imaging are clearly illustrated in the literature, and much can be determined over the phone. Simple plain film imaging and an accurate history often suffice for pre-operative planning with limited need for further FTF interactions following the initial consult.

Decreased number of patients needing a face-to-face consultation

The number of patients needing an FTF consultation decreased with the implementation of a clinician-led triaging system but was not shown to be statistically significant. One suggested reason is that the relatively small cohort of patients in 2022 could be skewed towards those with more complex presenting complaints needing FTF follow-ups. Nevertheless, the expertise and nuance of a clinician cannot be understated in the streamlining of telephone consultations for future use. Knowing which patients can be selected using experience is invaluable. There may be instances in which having an FTF meeting is unavoidable. Thorough initial FTF consultations would set the base examination findings, which would make further TFUs more successful and simpler to plan.

Cases for discussion drawn from the study

A successful case example of a telephone consult involves a 74-year-old gentleman who was an elective initial consult for superficial ulcers on the plantar aspect of his right foot, along the Achilles tendon. It was decided over the phone that the patient would need tendoachilles (TA) lengthening or gastrocnemius recession, given his symptoms and GP referral, and he was listed for the procedure. This case brings to light the effectiveness of telephone consultations for operative planning.

The case of a 47-year-old lady who suffered from bilateral hallux valgus deformity and bilateral Achilles tendinopathy had a telephone consultation as an elective follow-up patient. It was decided that she was to be admitted for Achilles debridement and excision of Haglund's deformity as well as reattachment, though her initial plan was to have bunion surgery. This highlights the usage of telephone consultations to alter operations and procedures, which would be based on the patient's symptoms and subjective pain rather than tactile examination, elements that can be simply assessed over the phone.

Circumstances for patients may change in the period before their previous appointment: for example, a 36-year-old lady who had originally suffered a talus bone bruise and partial anterior talofibular ligament (ATFL) tear of the left ankle was scheduled as a TFU patient. MRI showed partial ATFL tear and tibiotalar band with deltoid ligament. She was making steady progress till she wore high heels, which exacerbated the issue, and given her symptoms, she was offered FTF consultation in three weeks. When seen FTF, she was put on the list for a left ankle arthroscopy and reconstruction of the lateral ligament. She had to be swapped to an FTF appointment from TC following changes in the circumstances of her pathology.

The development of algorithms to choose which patients can be selected for TFU would likely involve not only the extent of injury but also the procedures being considered. This point was highlighted numerous times throughout the study. For example, an MRI scan of a 58-year-old man’s right foot displayed a 7-mm thickening of the medical limb plantar fascia. Shockwave treatment was discussed for this patient over the phone; the result of this was that the patient did not need treatment as his symptoms had improved. Furthermore, the patient did not need the steroid injection because he was pain-free.

Reduced number of patients who did not attend

Reduced DNAs is likely multifactorial and has a negative financial impact not only on the National Healthcare Service but also on the patient themselves as they are likely to represent their complaint further down the line with potentially worsened pathology [3]. Clinician-led triaging systems may provide patients more security in the decision made to have TC as opposed to FTF appointments. This results in stronger patient-clinician confidence, thereby increasing adherence to the system. Surveys were conducted across other outpatient clinics for those patients who did not attend; the findings suggest other factors such as patients simply "forgetting" that they had an appointment, and the patients favoured a text messaging reminder system to help reduce non-attendance. Almost half of the respondents said that they would be willing to pay a refundable booking fee [4]. Patients likely felt reassured before the TFU that they had already seen a clinician FTF during the initial consultation, therefore resulting in fewer DNAs for the follow-up period when compared to the initial consult data. The decision of which cohort of patients can safely be transferred to TFU is dictated by clinical judgement and works to prevent unnecessary workload associated with bringing the patients to the hospital.

The importance of clinician-led triage across different specialties and regions has been highlighted in a study by Burström et al., which compared the performance of different triage models across emergency departments in Sweden [5]. They found that the median length of stay was 158 minutes for physician-led team triage compared to 197 minutes for nurse/junior clinician triage, respectively (p < 0.001). The rate of patients leaving before the completion of treatment was reduced for clinician-led team triage compared to nurse/junior physician triage (p < 0.001). This study highlights that efficient clinical decision-making that accompanies the experience of a senior clinician improves the effectiveness in running healthcare provision runs. The clinician can quickly narrow differential diagnoses and choose only the investigations needed for the patient’s presenting complaints.

Increased number of investigations being requested using TFU

Cost-effectiveness is multifaceted and difficult to analyse in a single-centre trial. Previous literature in this field outlines the potential savings that are theoretically sound and the methods reproducible across different specialties. Akobeng et al. [6] conducted a randomised control trial of 86 paediatric patients with inflammatory bowel disease in Manchester Hospital (United Kingdom). This study found that telephone consultations cost £35.41 per patient on average compared to FTF consultations which cost £51.12 with a difference of £15.71 per consultation. The study reviewed the costs used including medical consultant-level salary (since all the consultations in this study were conducted by consultants), national insurance, overheads, training, and capital overheads. The study suggests further research to analyse the cost savings for patients with telecommunications, which would involve the patients using a personal diary of their costs with FTF compared to TFU. There are concerns regarding clinical effectiveness and limitations of virtual physical examinations as well as the potentially widening disparities in access [7]. Fink et al. conducted a randomised controlled trial of 123 general surgery patients in an outpatient clinic [8]. They showed that the mean consultation times were significantly shorter for telemedicine than FTF clinics (telemedicine: 10.52 ± 7.2 min, FTF: 15.95 ± 9.96 min, p = 0.0021) while maintaining acceptable patient satisfaction.

Patient satisfaction

A valid concern amongst clinicians and patients alike is the patient satisfaction aspect of telemedicine and whether a compromise between efficiency and properly addressing patients’ concerns can be reached [9]. Vusirikala et al. [10] conducted a single centre, prospective study in an NHS District General Hospital in May 2020, analysing patient satisfaction in an Outpatients Orthopaedic Clinic. Vusirikala et al. looked at patient satisfaction for 100 adult patients in addition to 25 clinicians. About 93% of overall patients were satisfied with telephone consultations, and 79% were willing to continue this method of consultation post-pandemic. One possible explanation for this was a decrease in patient’s time investment into the appointment, i.e., not having to alter their daily routine to accommodate the consultation. About 72% of clinicians reported overall satisfaction with this service, and 80% agreed that telephone consultations should be used in the future. Melian et al. [11] conducted a large cohort (n = 853) observational study in New Zealand and found that patients in the telephone group preferred teleconsultation over in-person office visits during the COVID-19 lockdown. Kaur et al. [12] conducted a literature search in June 2022 for patient satisfaction following a telephone consultation across seven countries. They concluded that most of the participants were found to be satisfied with the quality of telemedicine they were offered. The level of satisfaction was found highest amongst studies conducted in developed countries/states/cities such as New York City (94.9%), Los Angeles (82.7%), and the United Arab Emirates/UAE (81%).

Limitations of the study and future studies

Poor selection of patients for TFU may potentially increase the overall workload as they will then be required to be seen FTF. This article further highlights the importance of developing reproducible and validated scoring tools to standardise the selection of patients for TFU whilst having the flexibility to complement the clinician’s discretion. One proposed method is to analyse the demographics of patients called back for FTF versus TFU via multiple regression statistics to develop a multiparametric scoring tool. Understandably, this would be challenging to implement, given the complexity and variety of injuries presented in the clinic. If these confounding factors can be nullified with risk stratification scores, this would standardise the selection procedure and allow for automation in the selection process. The data collected in this article allows for future analysis of patient satisfaction with telemedicine and to take into account their suggestions. There are undoubtedly monetary benefits of telemedicine. Unfortunately, the quantification of the savings is difficult and beyond the scope of this paper. This study involves a single-centre trial associated with a relatively small sample size. The sample size is drawn from August to December for each cycle (2020 and 2022) in a single orthopaedic clinic to mitigate seasonal bias. Further research is needed within the field, which can be done by employing a variety of orthopaedic clinics in different geographical locations to further validate results regarding the outcomes of telemedicine.

Suggestions

The major drawback to teleconsultations is the lack of observable body language from both the patient's and the clinician’s perspective. Body language from patients can highlight painful areas that they may underplay for a variety of reasons. The Non-communicative Patient's Pain Assessment Instrument (NOPPAIN) criteria [13] emphasise the importance of** **highlighting facial expressions during the examination as body language tells a story that words cannot. Even though advancing artificial intelligence (AI) technology has shown promise in understanding these motions and cues in some cases, it leaves much work to be done to be comparable to an FTF understanding of body language afforded by clinician-patient interactions [14]. One suggested workaround for this is the implementation of video consultation. This would require the collaborative efforts of local and national service providers to ensure adequate supply and technical support for materials needed to make this a realistic option. Technical training for clinicians, patients, and administrative staff, along with software guidance and on-call support, would need to be firmly cemented before implementation. However, the benefits reaped would, in theory, be invaluable. Patients with difficulty in attending FTF can have their presenting complaints visualised, which is especially important in the orthopaedic setting. Clinicians would be able to share imaging with the patients and discuss their findings and reports, thereby undoubtedly increasing patient confidence in an ever-increasingly autonomous healthcare system.

Clinicians should be trained on how to conduct teleconsultations and how to alter the line of questioning to draw as much information as possible without the usual aid of tactile examination. Perhaps, video consultation and telecommunication as a short-term alternative will allow for good patient satisfaction and compliance [15,16]. As we progress beyond the scope of concern for COVID-19 and the theoretical need for social distancing decreases, it remains to be seen whether the general public begins to request a return to FTF appointments, sacrificing the logistical benefits of TFU for the warmth of the doctor-patient relationship. As there is a shift towards digital medicine [17], there will be inevitable doubts about losing the personal touch that medicine affords vulnerable patients. Scherer et al. surveyed consecutive regular orthopaedic and orthopaedic trauma patients at outpatient clinics from three European trauma centres [18]. The most common reason stated by 780 participants for opting to conduct a video consultation was "communication of medical findings." "No physical examination" was the most frequently stated disadvantage (75.9%). Participants who were above 55 years old were significantly less likely to use a remote consultation than their younger counterparts (OR = 0.18, p = 0.003). Within their cohort, it was concluded that elderly patients appear to be less eager regarding video consultations. Perhaps efficiency does not always translate to better medical care for every population.

## Conclusions

Whilst the future of telemedicine remains uncertain, it is apparent that there is movement towards a digitised platform underpinning medical practice. Telephone and video utilisation in modern medicine seems only natural in a world with fewer physical interactions in favour of practicality. It remains to be seen whether this plays into a long-term benefit. This article highlights the application of triaging systems for telephone consultations and the plethora of structural changes needed, if digitised medicine is to be widely implemented for day-to-day use. Cost analysis and multiple regression models to programme a scoring tool are areas needing further research across multiple centres with subsequent validation. It is recognised that certain groups of patients may be less familiar with digital communication. However, it becomes apparent that telemedicine offers a valuable service lifeline for vulnerable patients who are unable to attend hospital appointments, thereby improving patients’ quality of life and allowing their issues to be heard.
